# Deep viral blood metagenomics reveals extensive anellovirus diversity in healthy humans

**DOI:** 10.1038/s41598-021-86427-4

**Published:** 2021-03-25

**Authors:** María Cebriá-Mendoza, Cristina Arbona, Luís Larrea, Wladimiro Díaz, Vicente Arnau, Carlos Peña, Juan Vicente Bou, Rafael Sanjuán, José M. Cuevas

**Affiliations:** 1 Institute for Integrative Systems Biology (I2SysBio), Universitat de València-CSIC, 46980 Paterna, València, Spain; 2 Centro de Transfusión de la Comunidad Valenciana, Valencia, Spain; 3 Department of Informatics, Universitat de València, València, Spain; 4 Department of Genetics, Universitat de València, València, Spain

**Keywords:** Virology, Microbial genetics, Taxonomy, Evolution, Genetics, Microbiology

## Abstract

Human blood metagenomics has revealed the presence of different types of viruses in apparently healthy subjects. By far, anelloviruses constitute the viral family that is more frequently found in human blood, although amplification biases and contaminations pose a major challenge in this field. To investigate this further, we subjected pooled plasma samples from 120 healthy donors in Spain to high-speed centrifugation, RNA and DNA extraction, random amplification, and massive parallel sequencing. Our results confirm the extensive presence of anelloviruses in such samples, which represented nearly 97% of the total viral sequence reads obtained. We assembled 114 different viral genomes belonging to this family, revealing remarkable diversity. Phylogenetic analysis of ORF1 suggested 28 potentially novel anellovirus species, 24 of which were validated by Sanger sequencing to discard artifacts. These findings underscore the importance of implementing more efficient purification procedures that enrich the viral fraction as an essential step in virome studies and question the suggested pathological role of anelloviruses.

## Introduction

The increasing amount of information provided by metagenomics has accelerated the discovery of novel viruses, showing overwhelming viral diversity at all levels^1^. Viral metagenomics has been used to identify viral agents causing disease outbreaks or associated with specific symptoms^2,3^, to study the virosphere diversity^4–6^, and to address specific aspects of viral evolution^7,8^. Many of the newly discovered viruses are not associated with any disease and are consequently called “orphans”^9^. The family *Anelloviridae* provides the clearest example, since only one member of the genus *Gyrovirus* has been confirmed to cause disease in chickens^10^, despite an increasing number of anelloviruses being discovered in wild and domestic animals^11–15^. Three genera are known to produce chronic infections in humans: torque teno virus (TTV, *Alphatorquevirus*)^16^, torque teno mini virus (TTMV, *Betatorquevirus*), and torque teno midi virus (TTMDV, *Gammatorquevirus*). Indeed, anelloviruses constitute the most prevalent human-infective viruses^17^.

Little is known about the biology of anelloviruses because of the lack of appropriate cell cultures and animal models. However, it has been established that human anelloviruses are distributed worldwide and are frequently present in blood, feces, semen and urine^18^. Since the discovery of the first anellovirus^19^, the diversity of this family is constantly increasing as new members are identified. The family *Anelloviridae* currently encompasses fourteen genera, and the International Committee on Taxonomy of Viruses (ICTV) has subdivided TTV, TTMV, and TTMDV into 29, 12 and 15 species, respectively. Taxonomic classification is currently based on the analysis of the entire ORF1 nucleotide sequence, with pairwise nucleotide sequence identity cut-off values of 35% and 56% to define a species and a genus, respectively^20^.

Studies analyzing the blood virome of apparently healthy individuals have also revealed the presence of unknown viruses^21–23^, which is particularly relevant when considering blood transfusions or organ transplantation^24^. Anelloviruses occupy the largest fraction of the blood virome^25^. Based on previous studies^26–28^, we have used a protocol involving high-speed centrifugation, random RNA and DNA amplification, and massive sequencing of 120 pooled-plasma samples from blood donors in order to characterize viral diversity. The multiple displacement amplification (MDA)^29^ method was used for random amplification, which preferentially amplifies circular single stranded DNA but has been successfully used to detect RNA viruses in biological samples^29^. Additionally, since contaminant nucleic acids potentially causing misleading results were expected to be present along the purification protocol^30^, three blank controls were also used for eventual subtraction of the identified taxons.

## Results

### Strategy and overall sequence output

**Figure 1:**
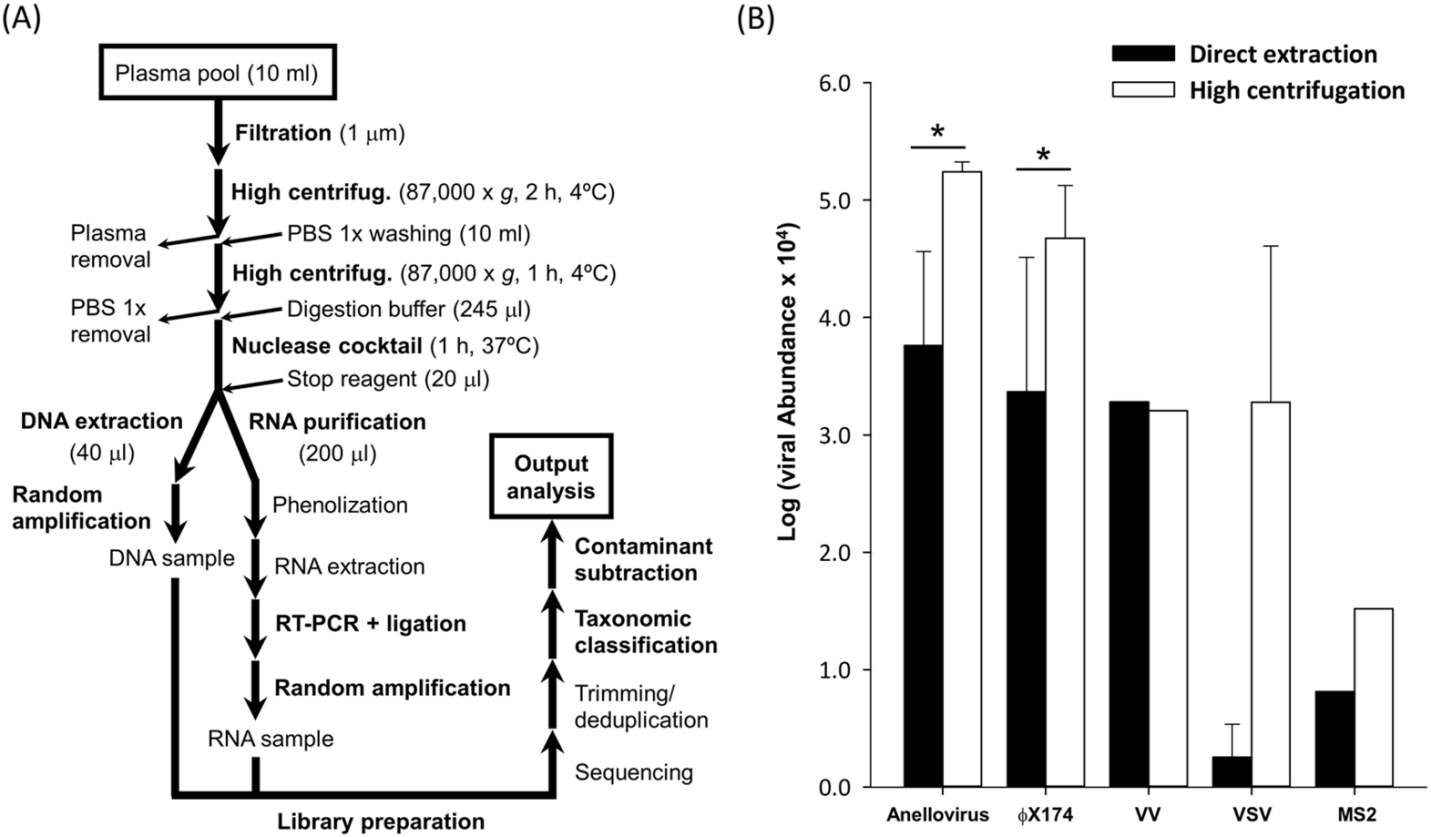
Experimental and bioinformatics workflow (**A**) and comparison between viral abundance estimated with direct extraction from plasma and the protocol involving initial high centrifugation (**B**). Main steps at panel (**A**) are marked in bold (See details in Methods section). For panel (**B**), comparison of normalized data was achieved by transforming total reads for each specific taxonomic group into abundance, which was obtained with Centrifuge using an Estimation-Maximization algorithm^31^ (See details in Methods section). For clarity, abundance × 10^4^ was represented in log scale. Error bars indicate standard error of the mean (SEM, *n* = 2 replicates). Asterisk indicates the statistical significance of a *t*-test analyzing the efficiency of the purification protocols (*$P<0.01$). For VV, the only indicated value for each treatment was obtained with 1 μm pore size filtration.

The protocol used in this study intended to enrich the viral fraction and different experimental combinations involving filtration and centrifugation steps were initially tested as explained below (Fig. 1A). Subsequent nuclease digestion for removing free nucleic acids was performed before independent viral DNA and RNA extraction, followed by random amplification and library preparation. Sequencing results were then taxonomically classified and taxons present in blank controls were subtracted. Since filtration and washing steps should select for viral particles, the vast majority of human and bacterial nucleic acids are expected to be removed or eventually subtracted in subsequent bioinformatics analysis.

We initially set out to compare viral recovery efficiency obtained by directly extracting nucleic acids from plasma or by performing a high-speed centrifugation step first. For this, we spiked a plasma pool, including ten individual plasma samples, with different viruses showing titers with biological meaning. Specifically, we spiked bacteriophages ϕX174 (non-enveloped, circular single-stranded DNA virus) and MS2 (non-enveloped, linear single-stranded RNA virus), vaccinia virus (VV, large enveloped, linear double-stranded DNA virus), and vesicular stomatitis virus (VSV, enveloped, linear single-stranded RNA virus), and used them for massive parallel Illumina sequencing (see details in “Methods” section). In this pilot study, we also analyzed anelloviruses (non-enveloped, circular single-stranded DNA viruses), since these are frequently found in blood. Two technical replicates differing in the pore size used at the initial filtration step (0.45 vs 1.0 μm) were performed, although this difference is only expected to affect large viruses such as VV^32^. Indeed, samples initially filtered with the largest pore size yielded thousands of VV reads, while VV was not detected in one of the samples filtered with the smaller pore size (Fig. 1B and Supplementary Table S1). When checking the presence of circular DNA viruses (anelloviruses and ϕX174), clear increases of viral recovery efficiency in terms of number of reads and abundance ranging one or two orders of magnitude were observed in the protocol involving a high-speed centrifugation step (Fig. 1B; *t*-test: *P* < 0.001 for both viruses). For VSV, only eleven reads were detected in one replicate of the direct extraction treatment, whereas thousands of reads were recovered when using high-speed centrifugation (Supplementary Table S1), these differences being marginally significant (*t*-test: *P* = 0.066). For MS2, viral reads were detected in a single replicate from each treatment and no clear conclusions could be drawn, although this could be accounted for by the low amount initially added for this virus. Despite the total amount of plaque-forming units (PFU) used for spiking MS2 and other viruses was the same, its low detection can be explained by the fact that it is an RNA virus with a small genome, and it is expected to be detected at lower levels than big DNA viruses (e.g. VV) or circular DNA viruses (e.g. ϕX174), which are preferentially amplified by MDA method. Consequently, since our results indicated that a high-speed centrifugation step substantially increased the recovery of circular DNA viruses and VSV, this approach was used thereafter in combination with an initial filtration step using a 1 μm pore size to avoid potential loss of large viruses. The sample obtained from ten pooled plasmas in this pilot study using the 1 μm filter and high-speed centrifugation was named pool 1 (P1). The samples subsequently obtained using these conditions, each including ten individual plasma samples, were named accordingly (P2–P12).

**Figure 2:**
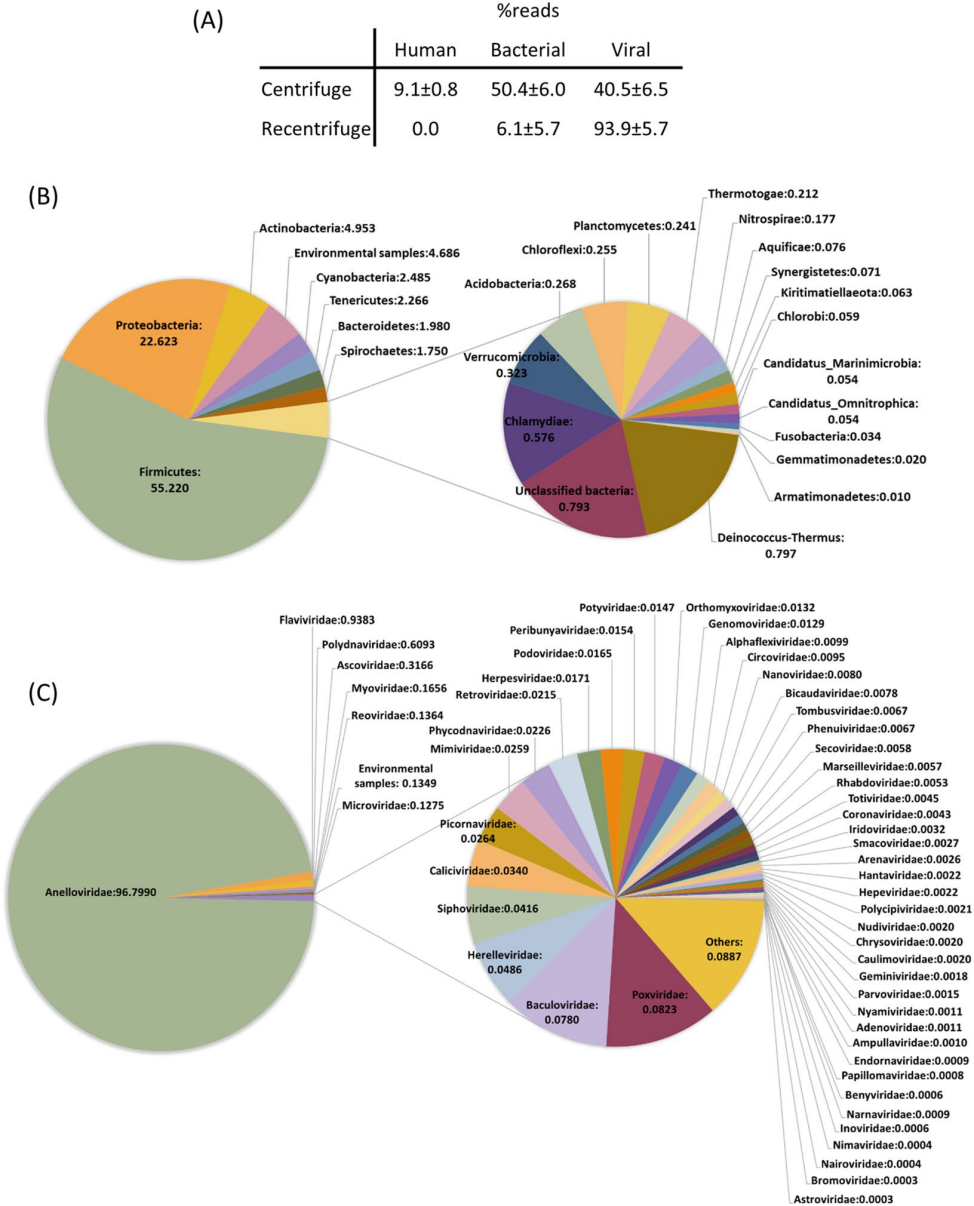
Summary of bioinformatics subtraction (i.e. mean frequency of reads (± SEM) before and after using Recentrifuge for the twelve analyzed pools) for human, bacterial and viral groups (**A**) and description of the microbiome (**B**) and the virome (**C**) characterized in this study. Classification is shown for bacteria and viruses at phylum and family level, respectively. Frequencies were obtained excluding spiked virus contribution in (**A**,**C**) panels.

Centrifuge software^31^ was used for taxonomic classification. The frequency of unassigned reads for each sample ranged between 0.9 and 6.8%. (Supplementary Table S2). A variable fraction of unassigned reads is commonly present in metagenomics studies analyzing virome composition^33–35^ and can be partially explained by reverse transcription and random amplification artifacts^36,37^. Since the purification protocol might carry over residual amounts of nucleic acids, it was essential to introduce blank controls to evaluate contamination risk. The reads obtained in these controls were used for taxonomic classification and subtraction of these potential contaminants from real samples using Centrifuge and Recentrifuge^38^ softwares, respectively. Then, we focused on reads belonging to taxons potentially present in our samples (i.e. human, bacterial and viral reads), although other taxonomic groups were also identified (Table 1 and Supplementary Table S2). We noticed that ambiguities in the taxonomical classification of reads were not properly handled by Recentrifuge, limiting our ability to remove potential contaminations corresponding to phylogenetically unclassified reads. Reassuringly, the total fraction of viral reads increased from 40.5 to 93.9% after the subtraction step (Fig. 2A). The total fraction of bacterial reads after subtraction dropped from 50.4% to 6.1%. As expected, human reads were removed by Recentrifuge. The non-removed bacterial reads encompassed 24 phyla (Fig. 2B and Supplementary Table S3), including Firmicutes (55.2%), Proteobacteria (22.6%), Actinobacteria (5.0%), Cyanobacteria (2.5%), Tenericutes (2.3%), and Bacteroidetes (2.0%). The relative proportions of these phyla are consistent with previous blood microbiome studies^39^, suggesting that these sequences may correspond to residual amounts of DNA that survived our virus-enrichment protocol. Alternatively, these could be contaminants that were not removed computationally.

**Table 1:** Summary of Recentrifuge results for the 12 pools analyzed.

Pool	# total reads	Bacterial reads	Anellovirus reads	Other viruses	Anellovirus contigs
P1	192,322	1704	147,618	1943	25
P2	125,327	4924	93,930	1106	9
P3	127,638	3538	111,188	1128	9
P4	150,324	8754	122,128	1859	4
P5	140,469	55,550	11,187	17,302	2
P6	35,120	4996	6132	2139	3
P7	368,499	6240	339,778	1057	22
P8	199,882	5303	171,517	4501	20
P9	536,796	5096	493,302	18,383	6
P10	167,112	5251	140,398	3851	8
P11	74,803	8505	25,269	943	2
P12	74,729	4514	44,321	3078	4

For each pool, the total number of reads passing Recentrifuge analyses, and those classified as bacterial, anellovirus and other viruses, are indicated. Last column shows the number of anellovirus contigs over 1.5 kb obtained in the assembling step. For clarity, viral reads from spiked viruses are excluded from counts.

Our samples contained sequences from 56 different viral families (Fig. 2C and Supplementary Table S4), but with a clear dominance of the *Anelloviridae* family, which represented 96.8% of the total fraction. The second most abundant family was *Flaviviridae* (0.92%), although most reads corresponded to a human pegivirus (HPgV) detected in pool 9 (16313 reads; genome coverage > 95%, average coverage depth 191×). This finding confirmed that our protocol was also efficient for RNA virus recovery. The remaining viral families were detected at lower frequencies, with read number ranging between 6 and 10,743. Potential human pathogens were not found on these families, suggesting that they are contaminants, which are commonly found in virome studies^30^. Indeed, most abundant families belonged to insect viruses (e.g. *Polydnaviridae* and *Ascoviridae* families, with 10,743 and 5582 reads, respectively) and bacteriophages (e.g. Caudovirales and *Microviridae* taxonomical groups, with 4801 and 2248 reads, respectively). It is worth mentioning that > 99% of reads assigned to the family *Circoviridae* were subtracted by Recentrifuge. The detection of members from this family has been associated with contaminated reagents^30^, which stresses the necessity of including appropriate controls. The fact that some reads from *Circoviridae* family still remain after bioinformatics subtraction with Recentrifuge can be a consequence of the technical limitations mentioned above.

### Analysis of HPgV

**Figure 3:**
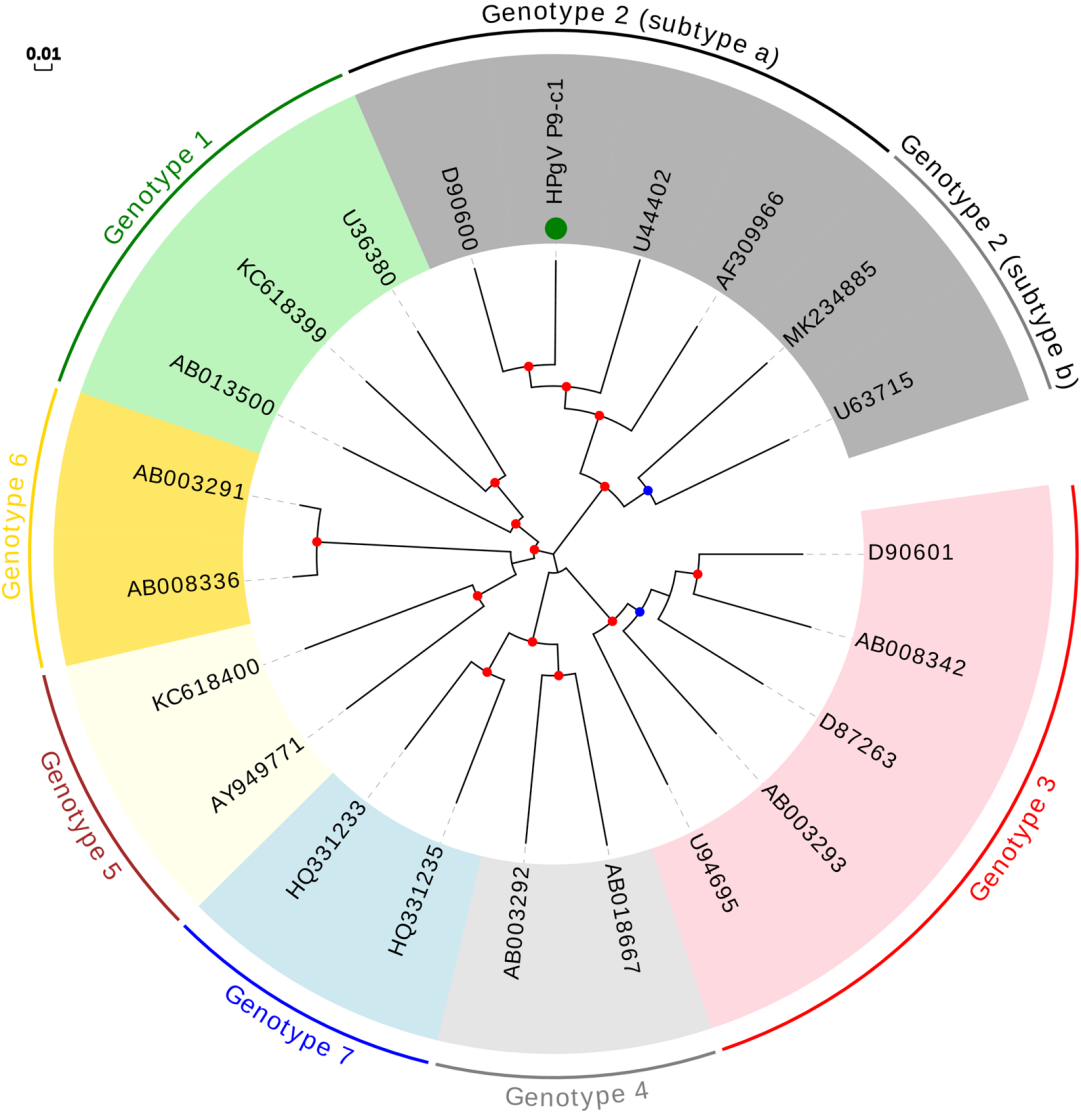
Phylogenetic tree based on the full coding sequence (i.e. the polyprotein) including representative isolates of the seven known HPgV genotypes. The sequence described in this study is indicated with a green circle. For genotype 2, distinct members from 2a and 2b subtypes are shown. Bootstrap values ranging 0.75–0.9 and 0.9–1.0 are indicated with blue and red circles, respectively. The scale bar indicates evolutionary distance in nucleotide substitutions per site.

Since the HPgV sequence detected in our study showed a high genome coverage, we decided to carry out a phylogenetic analysis for genotype assignation. To do this, we downloaded the nucleotide sequences of the complete polyprotein, which encompasses about 90% of the genome, from some representative isolates of the different known genotypes. The inspection of the phylogenetic tree (Fig. 3) allowed us to conclude that our sequence corresponded to genotype 2 and subtype a. This result is congruent with the geographic distribution of HPgV genotypes, since genotype 2 is commonly found in Europe and America^40^. Besides, the detection of a single isolate in our study is also consistent with previous studies showing that HPgV prevalence in developed countries ranged from 0.5 to 5%^41^.

### Analysis of anelloviruses

For each of the 12 pools, we generated contigs from all reads regardless their preliminary taxonomical classification, which avoided unintentional loss of viral reads and has recently been shown to be effective for detection of new anelloviruses^11^. Contigs larger than 1.5 kb were subsequently subject to Blast analysis. This showed that only a few contigs belonged to the viruses spiked in pools 1 and 2 or the above mentioned human pegivirus, whereas 114 contigs corresponded to anelloviruses, of which 23 showed overlapping ends and could thus be considered as complete genomes (Table 1 and Supplementary Table S5). Additionally, there was a significantly positive correlation between the number of contigs and the total amount of anelloviral reads in each pool (Spearman's correlation: *ρ* = 0.728; *P* = 0.004). We used the ORF1 nucleotide sequence for phylogenetic analysis. Full-length ORF1 was obtained for all but eight out of the 114 contigs (93%). For a preliminary taxonomic classification, we constructed a phylogenetic tree including ORF1 from Genbank hominid sequences (Supplementary Table S6), which allowed assignment of our contigs as belonging to TTV, TTMV or TTMDV genera (68, 29, and 17 sequences, respectively; Supplementary Table S5 and Supplementary Fig. S1). From the 23 contigs considered as complete genomes, 22 and one belonged to TTMV and TTMDV genera, respectively. Assembly efficiency was strongly affected by GC-rich regions present in anelloviruses, but these regions are shorter in TTMV genus^42^, which can facilitate full-length genomes completion. This also explained why several contigs fell into the expected full-length genome size range but did not present terminal redundancy.

**Figure 4:**
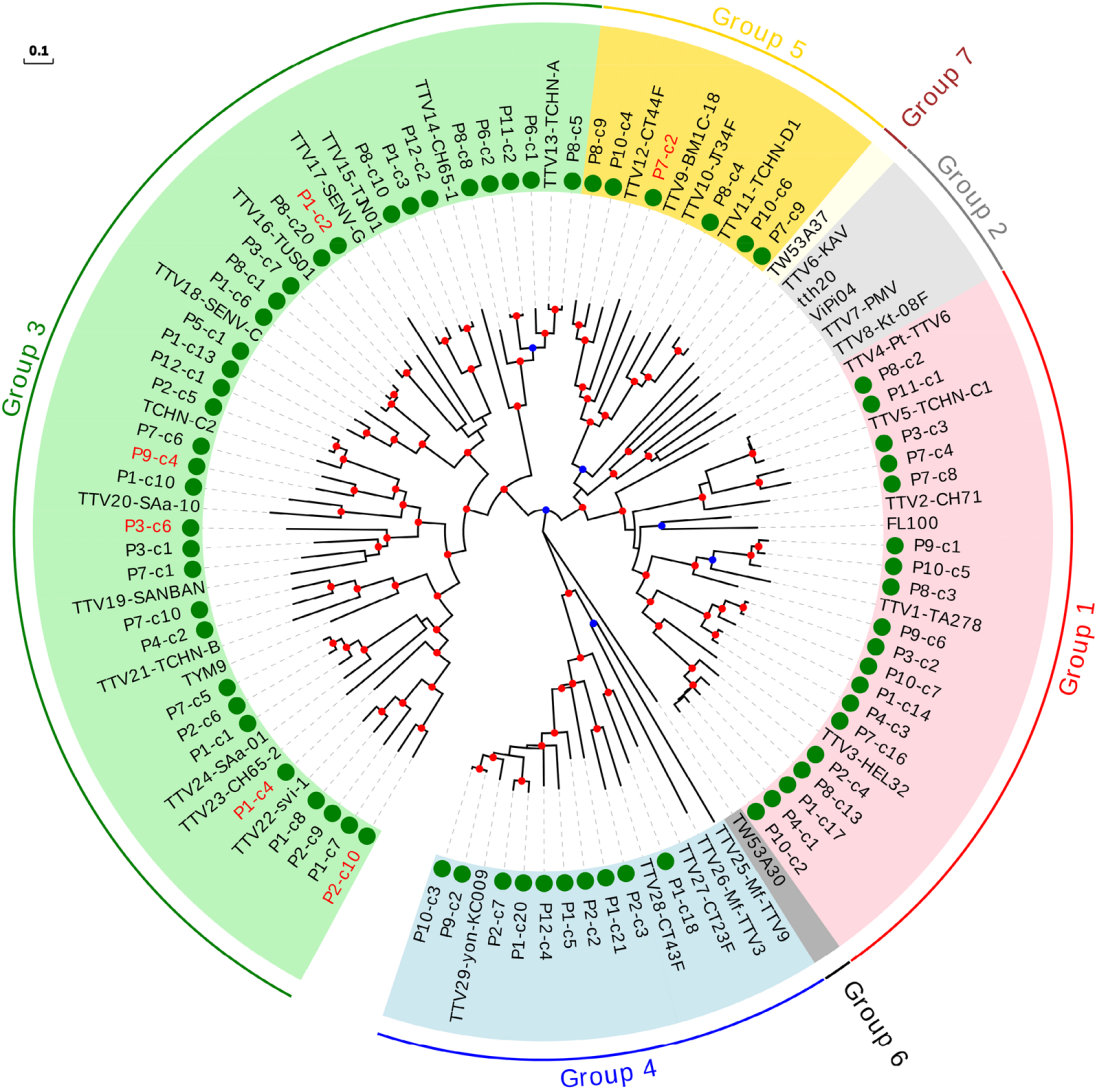
Phylogenetic tree for the ORF1 including the representative genotypes from TTV genus. Sequences described in this study are marked with a green circle. Those sequences that could be considered as new species are labelled in red. 0.7–0.85 and 0.85–1.0 bootstrap value ranges are indicated with blue and red circles, respectively. Scale bar indicates evolutionary distance in nucleotide substitutions per site.

In order to aid visualization, phylogenetic trees were independently constructed for each genus, and only one representative genotype of each species was used, including some that are not currently accepted by ICTV. For the TTV genus, which has been postulated to consist of seven phylogenetic groups^16^, the tree included our 68 new sequences as well as 36 previously described genotypes, each representing one known species (Fig. 4). This tree, along with divergence values, indicated that eight of our sequences could be considered as belonging to six novel species, whereas the remaining sequences clustered within 18 of the 36 previously known species (Supplementary Table S7). The number of our sequences assigned to each species was variable. For instance, four species clustered with only one of our sequences, whereas the species represented by genotypes TTV29-yon-KC009 and TTV3-HEL32 clustered with eight and ten of our sequences, respectively (Supplementary Fig. S2). This is in contrast with a previous study showing that TTV8 was the most quantitatively prevalent species in human blood^25^, as TTV8 did not cluster with any of our sequences. We also found no sequences that clustered with species belonging to groups 2, 6, and 7. However, there was a significant positive correlation between the number of species included in each group and the number of newly described sequences, even when discarding data from the recently proposed groups 6 and 7, which consisted of a single species (Spearman's correlation coefficient; $\rho =0.821$, $P=0.044$).

**Figure 5:**
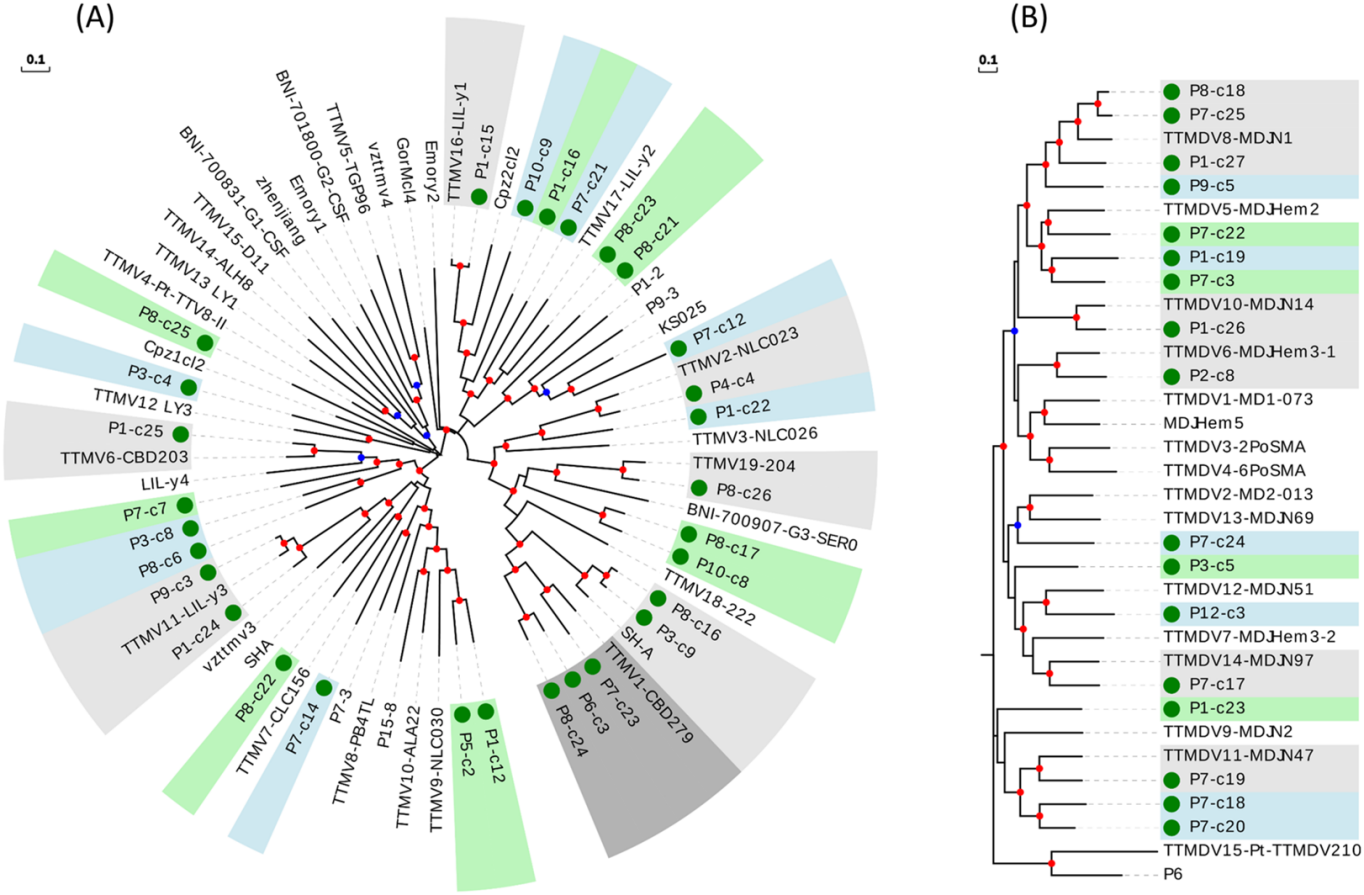
Phylogenetic trees for the ORF1 including the representative genotypes from TTMV (**A**) and TTMDV (**B**) genera. Sequences described in this study are marked with a green circle. New species (including one or more new sequences) are indicated with background green or blue color in order to distinguish contiguous clusters. Clusters of representative species including new sequences are indicated with background light or dark grey colors in order to distinguish contiguous clusters. 0.7–0.85 and 0.85–1.0 bootstrap value ranges are indicated with blue and red circles, respectively. Scale bar indicates evolutionary distance in nucleotide substitutions per site.

We then constructed a phylogenetic tree including 29 sequences from our study belonging to the TTMV genus and the 38 previously-described representative genotypes meeting the species demarcation criteria (Fig. 5A). Surprisingly, despite the smaller number of new TTMV sequences identified compared with the TTV genus (29 vs 68), most could be considered as novel species (Supplementary Table S8). In total, fifteen novel species were defined, three of which included two sequences, while the remaining 11 isolates clustered with seven of the 38 previously described species.

For the TTMDV genus, we identified 17 new sequences and used them to build a tree that also included 17 representative genotypes from known species. Similar to TTMV, we found that most new sequences are likely to correspond to novel species (Fig. 5B and Supplementary Table S9). Ten of our sequences defined 9 novel species, whereas the remaining 7 TTMDV sequences clustered with five of the 17 representative genotypes belonging to previously described species.

Assembly of massive sequencing reads could produce artifacts, eventually affecting the reliability of phylogenetic analysis. Since this possibility was particularly relevant for the assignation of new species, the DNA extracts from which we obtained 24 of the proposed new TTV, TTMV, and TTMDV species (4, 11, and 9 samples, respectively) were selected for reanalysis. These samples differed in average coverage depth, ranging from 4.9 × to 1747 × after assembly. For each, we performed sequence-specific PCR amplification and Sanger sequencing of the complete ORF1 (Supplementary Table S10). In all cases, Sanger sequencing confirmed the ORF1 sequences previously inferred by random amplification and Illumina sequencing, highlighting the reliability of our pipeline.

## Discussion

Implementation of large-scale blood virome studies is a powerful tool for the early detection of human emergent viruses causing chronic infections or exhibiting long asymptomatic phases, although surveillance programs based on this approach have not been established widely. Our results show that using adequate controls is essential in these studies, since contaminations can lead to false positives^43,44^. In our study, we have used three negative controls throughout the experimental protocol, and taxons eventually identified in these controls have been computationally subtracted from the samples. Since samples were initially filtered and used for digestion of free nucleic acids, we expected the non-viral fraction to be drastically reduced. Nevertheless, our data contained a significant fraction of bacterial and human reads, but these could be significantly reduced by bioinformatics subtraction. In our study, we have used MDA assay for random amplification, which can preferentially amplify circular single stranded DNA viruses^29^, such as anelloviruses. This amplification bias can partially explain the overwhelming presence of this family in our results, but sensitive detection of an RNA virus confirms the robustness of the proposed procedure.

Viral metagenomics should also benefit strongly from the implementation of procedures involving pre-amplification, purification, and enrichment steps as the one described here, since this increases sensitivity^27,28^. Indeed, this is supported by a recent study that analyzed anellovirus distribution in small mammals, in which sample purification involved sucrose gradient ultracentrifugation^11^. This study detected 11 potential novel species, and proposed the inclusion of two novel genera within the *Anelloviridae* family.

Together with previous studies, our result show that the diversity of anelloviruses is particularly remarkable in comparison with other viral families^9^. The fact that human and non-human primate isolates cluster phlylogenetically^45^ suggests that anelloviruses are an ancient family, and that the genetic diversity of this family is the consequence of millions of years of evolution. It has recently been proposed that the increasing amount of viral sequences identified by metagenomics should be incorporated into the ICTV classification scheme^46^. This inclusion, which should require appropriate quality control, is important for obtaining a more realistic picture of viral global diversity. Although this proposal is particularly relevant for environmental samples, the ICTV picture for *Anelloviridae* does not reflect its continuously increasing diversity.

Most of the sequences detected in our study belong to the TTV genus, which has been more extensively studied than other anellovirus genera. Yet, potentially novel species were mainly found among TTMV and TTMDV genera. It is likely that the later are more difficult to detect in protocols lacking viral enrichment and, hence, remain more poorly characterized. As such, our results underscore the importance of using viral enrichment methods for the study of anellovirus diversity.

It has been proposed that anellovirus load in blood increases in immunosuppressed patients, as has been described in transplanted^47^ and HIV-1 patients^48,49^. It has also been shown that anellovirus prevalence is lower in healthy subjects than in patients with common pathologies^50^. This has led to the suggestion that viral load could be used as a health biomarker in patients with chronic conditions, or even in people without known pathologies^51,52^. TTVs have also been postulated as biomarkers for anthropogenic pollution^53^, graft rejection^25^, and immune status^47^. However, cause-effect relationships between TTV load and health status need to be better clarified.

The prevalence of TTMV and TTMDV is markedly lower than that of TTV^25,50^. Overall, apart from some indirect evidence, viruses from the TTDMV genus have not been associated with pathologies^54^. In contrast, many of the recently described anellovirus species belonging to the TTMV genus have been associated to specific pathologies^55–58^. As a note of caution, associations between the presence of a virus and a pathological condition does not necessarily prove causality. As indicated above, anellovirus load could be a consequence of immune status. A lower load in healthier individuals could limit viral detection, leading to a statistical (but causal) association between the presence of a given virus and certain diseases. An illustrative example of this possibility is given by genogroup 2 from TTV, which has been detected at a very low frequency in the healthy population^59^. Sequencing and qPCR studies, including our results, have shown that genogroup 2 is absent or detected at low frequencies in healthy donors^60–62^, sporadically absent in transplanted patients^63^, and detected at higher frequencies in immunosuppressed patients^25,60,62,64^. In addition, it has also been shown that TTV viral load increases with the number of TTV genogroups simultaneously infecting a patient^59,60^, and that transplantation influences genogroup distribution^60^.

The metagenomics era has led to a new ecological perspective in virology, which avoids considering viruses necessarily as disease-causing pathogens^65^. Instead, viruses are regarded as integral components of ecosystems that can sporadically cause emerging diseases but also can be beneficial to their hosts^66,67^. Human anelloviruses, and probably most members of this family, seem to be essentially innocuous^17^. Indeed, potentially beneficial effects on human health have been suggested^9^. For instance, infection of newborns^68^ could promote the development and maturation of the immune system^17^. Besides, the detection of the same type of TTV in samples collected 16 years apart support the theory that people can remain chronically infected^69^. These results are in agreement with a long history of coevolution between the virus and the host, eventually leading to commensal or even mutualistic relationships.

## Methods

### Sample collection

A total of 120 plasma samples from healthy donors were collected from the Centro de Transfusión de la Comunidad Valenciana (Valencia, Spain) from September 15, 2018 to March 30, 2019. All samples were stored at − 80 °C until use. All subjects gave written consent in accordance with the declaration of Helsinki. The protocol was approved by the University of Valencia ethics committee (IRB No. H1489496487993). Plasma samples were divided into 12 heterogeneous pools in age and gender (each pool included ten samples, Supplementary Table S11).

### DNA/RNA extraction and amplification

For a pilot study, an initial pool of 10 samples was obtained (P1 pool) by mixing 2.5 mL of plasma from each sample into one tube (25 mL total). To assess viral recovery, we spiked this pool with 10^3^ PFU/mL of ϕX174, vaccinia virus (VV) and MS2, and 10^4^ PFU/mL of vesicular stomatitis virus (VSV). Half of the total volume was then filtered through a 0.45 μm or a 1.0 μm filter to remove cells and other non-viral particles. Since this different filtration is only expected to compromise the detection of big viruses^32^, both filtered fractions could be considered technical replicates for all spiked viruses except VV. From each fraction, 1 mL was used to extract nucleic acids with the QIAAMP Ultra Sens Virus Kit (Qiagen) following the manufacturer’s instructions. DNA was amplified from the final elution with the TruePrime WGA kit (Sygnis), whereas RNA from half of the final elute volume was cleaned with TRIzol LS reagent (Invitrogen), extracted with the QIAamp Viral RNA Mini kit and amplified using the QuantiTect Whole Transcriptome kit (Qiagen), which includes a ligation step following reverse transcription. In parallel, 10 mL from each filtered fraction was subject to high-speed centrifugation (87,000*G*, 2 h, 4 °C), washed with PBS 1X (87,000*G*, 1 h, 4 °C) and resuspended in 245 μL 1X digestion buffer (Turbo DNA Free kit, Ambion). Then 5 μL of Turbo DNase, 2 μL of Benzonase (Sigma) and 2 μL of micrococcal nuclease (NEB) were added to the sample to remove unprotected nucleic acids. After incubation (1 h, 37 °C), 20 μL of stop reagent was added, following the manufacturer’s instructions. Then, 240 μL supernatant was transferred to a new tube and split into two fractions: 200 μL fraction was used for RNA extraction and final amplification as previously described, and 40 μL fraction was used for DNA extraction with the QIAamp Viral RNA Mini kit and amplification with the TruePrime WGA kit.

For the other eleven groups (P2-P12 pools), mixes were done adding 1 mL plasma from each sample. As a control, 5 × 10^2^, 10^3^ and 10^4^ PFU of ϕX174, MS2 and VSV were added, respectively, to pool 2. Three blank samples starting the whole extraction protocol from 10 mL PBS 1X were used for subtraction of potentially contaminant taxons.

### Massive parallel sequencing

For the pilot study, the 8 amplification products obtained (2 replicates × 2 extraction methods, direct extraction versus high-speed centrifugation × 2 types of products, DNA/RNA) were used for library preparation using Nextera XT DNA library preparation kit with 15 amplification cycles (Illumina) and sequenced using a MiSeq device. For the rest of the pools, DNA and RNA amplification products were mixed in equimolar concentration before library preparation and sequenced in a NextSeq device. The raw sequence reads from the metagenomic libraries were deposited in the Short Read Archive of GenBank database under accession number PRJNA691135.

### Sequence analysis

Sequence data was quality checked using FastQC v0.11.8 (http://www.bioinformatics.babraham.ac.uk/projects/fastqc/) and MultiQC v1.8^70^. Reads were first deduplicated using clumpify.sh and then quality filtered using bbduk.sh, both from BBTools suite v38.68^71^. A quality trimming threshold of 20 was used and reads below 70 nucleotides in length were removed from the dataset.

The metagenomics analysis was carried out using the Centrifuge software package^31^ version 1.0.4 using a minimum exact match of 18. A customized database was generated from the NCBI nt database downloaded in June 2019. The Centrifuge download tool was used to incorporate archaea, viruses, bacteria and fungi genomes from the NCBI RefSeq database. Finally, draftGenomes^72^ was used to supplement the database with the SMS sequences in the NCBI WGS database belonging to viral taxa. Centrifuge results were postprocessed for contaminant removal and analyzed with Recentrifuge^38^ version 1.1.0 using a minscore of 22. In addition to per-read classification, Centrifuge was also used to perform abundance analysis for the spiked viruses and anellovirus family from the pilot study. To do this, Centrifuge uses a statistical model to find maximum likelihood estimates of abundance through an Estimation-maximization algorithm^31^.

Assembly was individually performed for each pool with SPAdes^73^ version 3.14.0 using default parameters. Homology analysis of the contigs was performed against a local copy of the NCBI nucleotide (nt) database using BLASTn v2.10.0 with an E-value cutoff of < 10^–5^. For each anellovirus contig, average coverage depth was estimated using bbmap.sh from BBTools suite v38.68. Anellovirus contig sequences and the new HPgV sequence were deposited in GenBank under accession numbers MW455345-MW455458 and MW467971, respectively.

Putative open reading frames were identified using ORF Finder (https://www.ncbi.nlm.nih.gov/orffinder/).

### Phylogenetic analysis

To study phylogenetic relationships in *Anelloviridae* family, nucleotide ORF1 sequences from hominid TTV, TTMV, and TTMDV isolates available from Genbank by February 2020 were downloaded (Supplementary Table S6). Regarding HPgV phylogenetic analysis, nucleotide sequences for the complete polyprotein from representative isolates of all currently known genotypes were downloaded. Sequence alignment (on the basis of the amino acid sequences) was performed with MUSCLE^74^ as implemented in MEGA version X^75^ and subsequent phylogenetic inference using nucleotide sequences was conducted with the maximum likelihood (ML) method also implemented in MEGA version X. Analysis were performed under the best fit nucleotide substitution model identified as GTR + Γ + I using Akaike information criterion as the model selection framework in MEGA version X. The reliability of the phylogenetic results was assessed using 1000 bootstrap replicates. The final trees were annotated with EvolView^76^. Anellovirus species demarcation was performed by checking nucleotide pairwise identity matrices obtained independently for each genus.

### Sanger sequencing

Sequence data obtained from assembled contigs for several anelloviruses were used to design primers amplifying the complete ORF1. Then, 25 μL PCR reactions were performed adding 1 μL DNA, Phusion High-fidelity DNA polymerase (ThermoFisher Scientific) and GC buffer using specific annealing conditions for each amplification product. PCR and additional internal primers were used for Sanger sequencing (Supplementary Table S10).

## Supplementary Information

Supplementary Information 1.

Supplementary Information 2.

Supplementary Information 3.

Supplementary Information 4.

Supplementary Information 5.

Supplementary Information 6.

Supplementary Information 7.

Supplementary Information 8.

Supplementary Information 9.

Supplementary Information 10.

Supplementary Information 11.

Supplementary Information 12.

Supplementary Information 13.

Supplementary Information 14.
