# Supplementary material for: Deep viral blood metagenomics reveals extensive anellovirus diversity in healthy humans
Source: Sci Rep. 2021 Mar 25;11:6921. doi: 10.1038/s41598-021-86427-4 (PMC7994813; doi:10.1038/s41598-021-86427-4)
Supplement: Supplementary file 1 — Supplementary Information 1. [file 41598_2021_86427_MOESM1_ESM.docx]

**Supplementary Table S1. Centrifuge results of the pilot study.** For each extraction (Direct extraction vs. high centrifugation) and filtration (0.45 μM vs. 1.0 μM) treatment, sequencing libraries were prepared independently for DNA and RNA samples. For spiked viruses and anelloviruses, number of reads and abundance is shown.

**Supplementary Table S2. Summary of Centrifuge results for the 12 pools analyzed.** For each pool, the total number of reads passing filtering/trimming analyses, and those classified as human, bacterial, anellovirus, other viruses, metazoa, viridiplantae, fungi and archaea are indicated.

**Supplementary Table S3. Results of bacterial taxonomic classification using Centrifuge for controls and samples.** Recentrifuge values are also provided for samples. Reads are provided at phylum, class and order level.

**Supplementary Table S4. Results of viral taxonomic classification using Centrifuge for controls and samples.** Recentrifuge values are also provided for samples. The number of total, eukaryotic, human, bacterial and viral reads is shown. Subsequent rows show the distribution of reads in the viral fraction, including spiked viruses, viral families and other taxonomic levels related with unclassified viruses.

**Supplementary Table S5. List of sequences/contigs detected in our study with the SPAdes analysis.** For each sequence, names in the first column refers to pool (P) and isolate number (c). Contig length (in nucleotides), average coverage depth, deduced size of the putative ORF1 (in amino acids), and accession number is given. Last column indicates genus assignment in accordance with phylogenetic and blast analyses. *contigs showing terminal redundancy and, consequently, considered complete genomes. **contigs yielding incomplete ORF1, since nucleotide sequence is interrupted before reaching initiation/stop codon.

**Supplementary Table S6. List of anellovirus isolates downloaded from Genbank.** Accession number, isolate name and anellovirus genus is indicated. *Those meeting the species demarcation criteria were chosen for subsequent phylogenetic analyses including the sequences described in our study.

**Supplementary Table S7. Nucleotide pairwise identity matrix obtained using ORF1 alignment of downloaded TTV representative genotypes and the new viral sequences assigned to this genus.**

**Supplementary Table S8. Nucleotide pairwise identity matrix obtained using ORF1 alignment of downloaded TTMV representative genotypes and the new viral sequences assigned to this genus.**

**Supplementary Table S9. Nucleotide pairwise identity matrix obtained using ORF1 alignment of downloaded TTMDV representative genotypes and the new viral sequences assigned to this genus.**

**Supplementary Table S10. Primers used for PCR amplification and Sanger sequencing of ORF1 gene.** Internal primers (F2/R2) were also used for sequencing when necessary.

**Supplementary Table S11. Demographic information of plasma donors included in each pool.**

**Supplementary Figure S1. Global phylogenetic tree for the ORF1 of the three anellovirus genera.** All downloaded sequences and the sequences described in this study (labelled in red) are included. 0.7-0.85 and 0.85-1.0 bootstrap value ranges are indicated with blue and red circles, respectively. Scale bar indicates evolutionary distance in nucleotide substitutions per site.

**Supplementary Figure S2.** **Phylogenetic tree including the representative genotypes from TTV genus.** The sequences described in this study that can be considered as new species are also included and labelled in red. Numbers between brackets indicate the number of new sequences clustering with a specific genotype (All new sequences are explicitly shown in Fig. 4). Bootstrap values are indicated in red at each branch point. Scale bar indicates evolutionary distance in nucleotide substitutions per site.
