# Supplementary material for: Deep viral blood metagenomics reveals extensive anellovirus diversity in healthy humans
Source: Sci Rep. 2021 Mar 25;11:6921. doi: 10.1038/s41598-021-86427-4 (PMC7994813; doi:10.1038/s41598-021-86427-4)
Supplement: Supplementary file 2 — Supplementary Information 2. [file 41598_2021_86427_MOESM2_ESM.docx]

|  | DNA libraries | | | | RNA libraries | | | |
| --- | --- | --- | --- | --- | --- | --- | --- | --- |
|  | Direct extraction | | High centrifugation | | Direct extraction | | High centrifugation | |
|  | 0.45 μm | 1.0 μm | 0.45 μm | 1.0 μm | 0.45 μm | 1.0 μm | 0.45 μm | 1.0 μm |
| # total reads | 2,468,272 | 2,774,265 | 797,932 | 835,504 | 3,183,681 | 1,875,367 | 3,002,159 | 2,456,356 |
| # classified reads | 2,369,737 | 2,736,761 | 784,395 | 827,987 | 2,979,637 | 1,778,578 | 2,662,271 | 2,230,725 |
| Human reads | 1,712,841 | 1,866,641 | 14,676 | 14,363 | 10,557 | 5,951 | 34,304 | 21,730 |
| Bacterial reads | 13,134 | 14,308 | 42,289 | 55,632 | 1,131,143 | 596,368 | 807,777 | 648,431 |
| VV reads/Abundance | 152/0.064 | 5,229/0.1908 | 0 | 1,338/0.1605 | 0 | 0 | 0 | 0 |
| φX174 reads/Abundance | 7,042/0.3114 | 4,199/0.1532 | 41,013/5.1971 | 35,722/4.2841 | 0 | 0 | 0 | 0 |
| VSV reads/Abundance | 0 | 0 | 0 | 0 | 11/0.0004 | 0 | 7,446/0.2670 | 2,598/0.1129 |
| MS2 reads/Abundance | 0 | 0 | 0 | 0 | 20/0.0007 | 0 | 92/0.0033 | 0 |
| Anellovirus reads/Abundance | 16,601/0.6984 | 12,412/0.4528 | 135,327/17.1483 | 147,618/17.7035 | 0 | 0 | 0 | 0 |
| Other viruses | 2,430 | 7,015 | 6,823 | 11,972 | 12,370 | 9,197 | 9,850 | 7,031 |
