# Supplementary material for: Deep viral blood metagenomics reveals extensive anellovirus diversity in healthy humans
Source: Sci Rep. 2021 Mar 25;11:6921. doi: 10.1038/s41598-021-86427-4 (PMC7994813; doi:10.1038/s41598-021-86427-4)
Supplement: Supplementary file 3 — Supplementary Information 3. [file 41598_2021_86427_MOESM3_ESM.docx]

| Pool | # total reads | % classified reads | Human reads | Bacterial reads | Anellovirus reads | Other viruses | Metazoa | Viridiplantae | Fungi | Archaea |
| --- | --- | --- | --- | --- | --- | --- | --- | --- | --- | --- |
| P1 | 835,504 | 99.1 | 14,363 | 55,632 | 147,618 | 49,032 | 205,681 | 59,302 | 12,214 | 1,928 |
| P2 | 1,948,103 | 96.0 | 55,003 | 237,092 | 126,117 | 24,170 | 544,766 | 270,667 | 69,736 | 2,480 |
| P3 | 1,008,317 | 96.6 | 26,916 | 120,081 | 117,659 | 19,515 | 265,499 | 125,575 | 25,382 | 2,995 |
| P4 | 1,909,824 | 95.7 | 43,516 | 230,218 | 139,537 | 22,437 | 518,405 | 256,947 | 52,663 | 4,445 |
| P5 | 2,420,824 | 96.6 | 33,118 | 355,178 | 17,461 | 42,128 | 720,739 | 402,583 | 41,911 | 4,787 |
| P6 | 2,706,682 | 93.2 | 48,748 | 328,786 | 7,882 | 5,744 | 1,050,021 | 278,520 | 74,294 | 1,996 |
| P7 | 1,771,038 | 95.6 | 41,209 | 181,139 | 339,778 | 16,529 | 428,762 | 217,249 | 35,940 | 3,028 |
| P8 | 1,712,606 | 95.5 | 43,062 | 191,273 | 188,955 | 32,940 | 458,886 | 199,406 | 42,769 | 4,524 |
| P9 | 2,042,540 | 95.8 | 34,865 | 215,035 | 493,302 | 34,481 | 373,521 | 268,978 | 38,457 | 3,065 |
| P10 | 1,541,190 | 95.7 | 46,055 | 169,011 | 155,854 | 13,926 | 391,019 | 230,288 | 27,651 | 4,127 |
| P11 | 2,390,182 | 95.6 | 45,404 | 349,863 | 29,247 | 28,671 | 565,804 | 353,213 | 76,877 | 4,078 |
| P12 | 1,935,479 | 94.9 | 47,649 | 224,878 | 60,981 | 23,457 | 554,726 | 318,607 | 64,112 | 4,001 |
