# Supplementary material for: Deep viral blood metagenomics reveals extensive anellovirus diversity in healthy humans
Source: Sci Rep. 2021 Mar 25;11:6921. doi: 10.1038/s41598-021-86427-4 (PMC7994813; doi:10.1038/s41598-021-86427-4)
Supplement: Supplementary file 11 — Supplementary Information 11. [file 41598_2021_86427_MOESM11_ESM.docx]

| **Isolate name** | **Primer name** | **Sequence (5’-3’)** | **PCR fragment size (nt)** | **Average coverage depth** |
| --- | --- | --- | --- | --- |
| P1-c2 | P1-C2F | GTTATCACTTCGACCCGCCGGCAC | 2427 | 291.58 |
|  | P1-C2R | CTCGTACTCACACTGCCAGTCCTCCTT |  |  |
|  | P1-C2R2 | CCTCCTTAGTGGGAGGACGA |  |  |
|  | P1-C2F2 | CCAAGGCCAGCACTGAGG |  |  |
| P1-c4 | P1-C4F | CAGAGCCTAACCCCGAGCAGCCATG | 2430 | 20.20 |
|  | P1-C4R | GGGGTAGTAAGGCCTGTCTGTGTGGTT |  |  |
|  | P1-C4F2 | CACATTAGAAGACTGGCTGTACAC |  |  |
| P1-c16 | P1-C16F | GCGGTTGCGATTCTACTATTTGCCACTT | 2143 | 943.28 |
|  | P1-C16R | CAGCTTCTGTTTGTTCTTCCCATGGATA |  |  |
| P1-c23 | P1-C23F | CCTGACCATCCAGACAGACAACTT | 2059 | 506.74 |
|  | P1-C23R | TTCTGTTTGTAGTTCAAAGCCTGGT |  |  |
| P3-c4 | P3-C4F | CCTACTGCTTCACATCTTTTCTCACGGAG | 2200 | 21.63 |
|  | P3-C4R | AGAGGTTCAGGTACAAGCCAGTCATAGT |  |  |
|  | P3-C4F2 | GACTACCAACGTGGAACAGCAC |  |  |
| P3-c5 | P3-C5F | TGGTGGAGAAGAAGAAACAAACCATGGTACT | 1993 | 82.00 |
|  | P3-C5R | GGCTCTACTCTAAAATTCCCGTTTGTAGT |  |  |
|  | P3-C5F2 | CCATTGCAACTGGCTCTGCAG |  |  |
| P3-c6 | P3-C6F | AGTCCAGACAACAATAACCCGAGAC | 2350 | 310.90 |
|  | P3-C6R | TGCATATTCTGTTTCCCAGTCTCCT |  |  |
|  | P3-C6F2 | CCAGCTTTTGGCAGTCAAATATAGCAA |  |  |
| P3-c8 | P3-C8F | TTGTGAATGTGAAAAACCACTTCAA | 2214 | 1540.48 |
|  | P3-C8R | AAAACAAATCATCTGCCCTTTCTTG |  |  |
|  | P3-C8F2 | GCAGCTCCAGGACTACTAAAACCAGG |  |  |
| P5-c2 | P5-C2F | ACCCACACCGCTACTTTTATCTTTGAACA | 2189 | 4.86 |
|  | P5-C2R | CCTCAAATTCTCTAGCAGTCATACGTCT |  |  |
|  | P5-C2R2 | GGACAAATACCAACCACAAACCTAC |  |  |
| P7-c3 | P7-C3F | CAACCATCCTTTTGCTCATTTGCTTGCT | 2269 | 322.59 |
|  | P7-C3R | AGGCTAAAGTTGACATAGGGTTCAGGGG |  |  |
|  | P7-C3F2 | TCTGCAGCTGACTTTAGACACCCAACA |  |  |
| P7-c14 | P7-C14F | CTACCAACCATTAGCACATACAGCAAC | 2211 | 7.95 |
|  | P7-C14R | CTTGTTCATATTCTTCAGGAGTCATTCTCC |  |  |
|  | P7-C14F2 | GCACAACAATATGGATATGGCACAG |  |  |
| P7-c18 | P7-C18F | TAATCACCAGAGACTACCAACAATGCCA | 2079 | 232.97 |
|  | P7-C18R | CCTTGGTGGTCTGTGAAATGCTG |  |  |
|  | P7-C18F2 | CTACTGGTTGGTTTCAACCAGAC |  |  |
| P7-c20 | P7-C20F | AAGTAGGTGAGCAAAGGGTAAGTTACA | 2081 | 12.33 |
|  | P7-C20R | TGGTCACAGCGATCGAGAATTAACTG |  |  |
| P7-c21 | P7-C21F | GCTGCAACGACACCACTCTACATA | 2119 | 485.28 |
|  | P7-C21R | CAGGGGTAAATCGTCTAGGTGGTC |  |  |
| P7-c22 | P7-C22F | CTGCTTGCCAATATCTTTCCTGTG | 2121 | 1747.00 |
|  | P7-C22R | TCACGTTCTGTGTCTTCTTCAAAGC |  |  |
|  | P7-C22F2 | AACAGCCTGCACATTTAGATACCCT |  |  |
| P7-c24 | P7-C24F | ATTTTTCCACTTGGCCATTCTGAT | 2086 | 392.27 |
|  | P7-C24R | TTCTGTGTCTTGTTCAAATCCTGGT |  |  |
|  | P7-C24F2 | CTGATTCATACAGAAAACCAAGCTC |  |  |
| P8-c17 | P8-C17F | GCAACTATTAACTGCCACGATCTCTGCT | 2109 | 459.02 |
|  | P8-C17R | TCAGCCTCCCAACTCTTTCCCCTTATAG |  |  |
|  | P8-C17R2 | CCAGTTTGTGGATACCAGTGCAG |  |  |
| P8-c22 | P8-C22F | TCCCATCTTAGATTTACAACTGAGGAA | 2166 | 468.96 |
|  | P8-C22R | TGTCTGGGATAGTGGTCCCATATTT |  |  |
|  | P8-C22F2 | GGAAACAGCAATGCACAAAGTTTAACC |  |  |
| P8-c23 | P8-C23F | AACTACTGGCAATATCCAACAAGCA | 1796 | 139.78 |
|  | P8-C23R | TAACTCTCTCTCATGGGGTGCAAAG |  |  |
| P8-c25 | P8-C25F | CACTGCAAATCACCTTTAAACCACA | 2109 | 383.53 |
|  | P8-C25R | CATGGTTTCATGCGTCTGTTTTTAG |  |  |
|  | P8-C25F2 | CAGAAAGCAGCACAATAACATTACATACCC |  |  |
| P9-c4 | P9-C4F | TCCCGCTCCTGATTCCCAAAACAATCC | 2442 | 38.09 |
|  | P9-C4R | AGGGGTAGTATGGAGTGTCTGACAGGT |  |  |
|  | P9-C4F2 | AGACTTCTATTACCCCATAATCGG |  |  |
| P9-c5 | P9-C5F | TTTTCCTCCTGGCCATCAAGACAG | 2140 | 19.66 |
|  | P9-C5R | GGGGTGTCCTCTTTAAACAATCTAGGGG |  |  |
| P10-c9 | P10-C9F | GGATTCCAAAAACCCGAAACAGATA | 2122 | 36.15 |
|  | P10-C9R | TTGTTGTTCCCAGGGTGTTAGTCTT |  |  |
|  | P10-C9F2 | GATACAATACACACACATGCAGACTC |  |  |
|  | P10-C9R2 | GTTCATATCTTCGTATGGGTCAAAG |  |  |
| P12-c3 | P12-C3F | TGCTTGCCAGTATCTTTCCTGTGGGTCA | 2149 | 13.63 |
|  | P12-C3R | CAGGTCTGAGAAATGCAAAAGCAAGTTCA |  |  |
|  | P12-C3F2 | TGCCCAGAAGCAACTTACACATAC |  |  |
