# Supplementary figures and images for: Deep viral blood metagenomics reveals extensive anellovirus diversity in healthy humans
Source: Sci Rep. 2021 Mar 25;11:6921. doi: 10.1038/s41598-021-86427-4 (PMC7994813; doi:10.1038/s41598-021-86427-4)

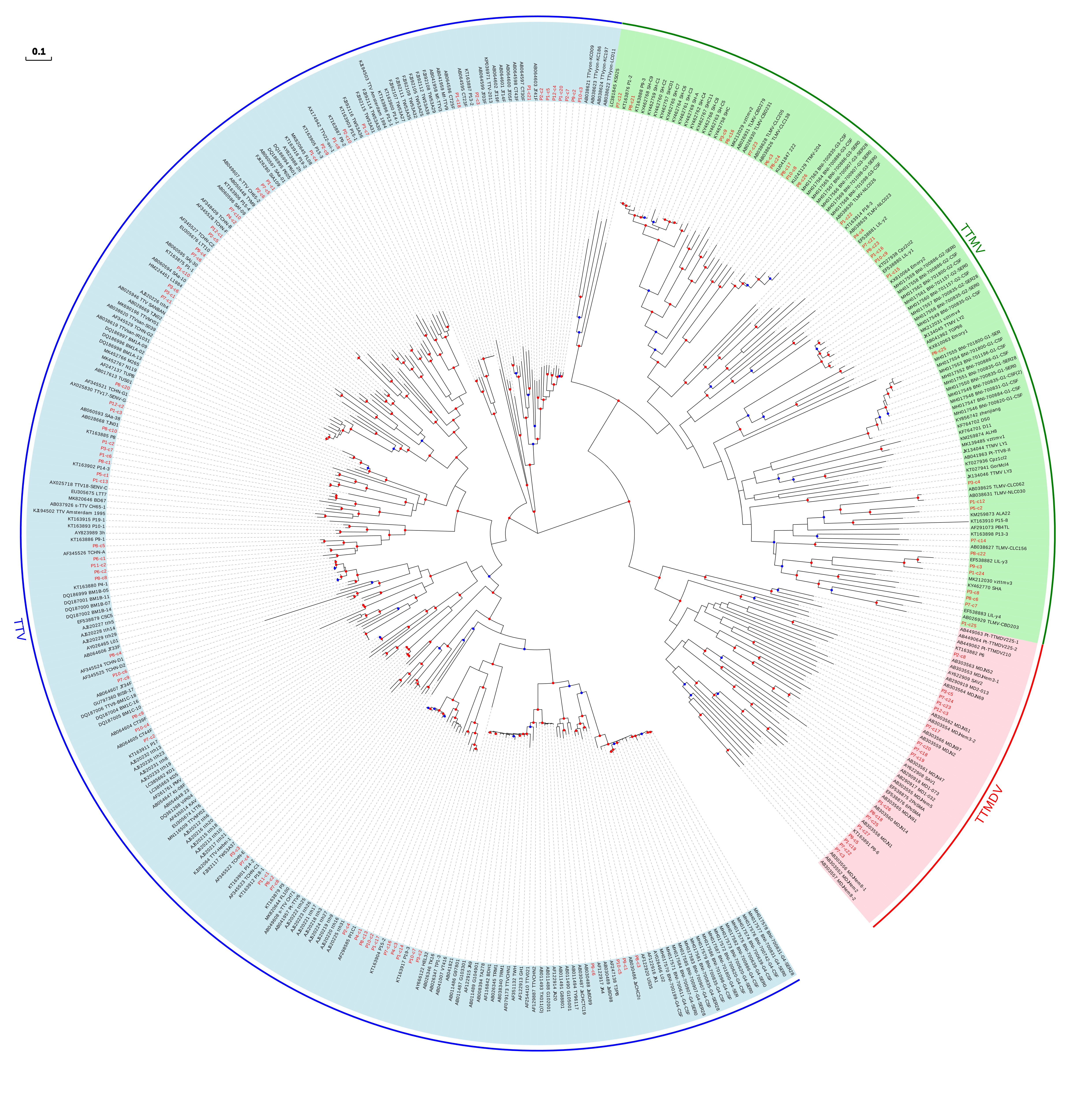

Supplement: Supplementary file 13 — Supplementary Information 13. [file 41598_2021_86427_MOESM13_ESM.tiff]

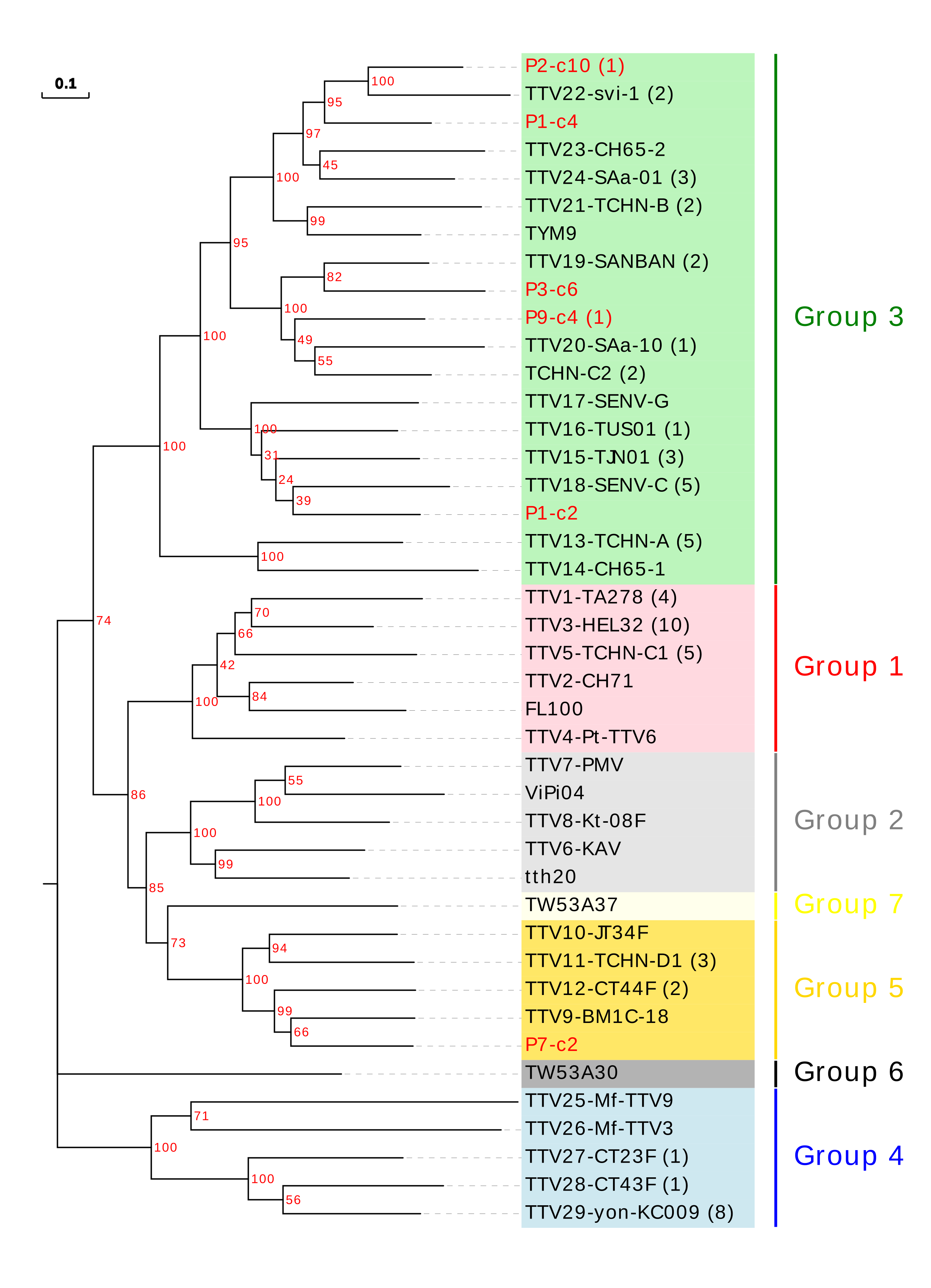

Supplement: Supplementary file 14 — Supplementary Information 14. [file 41598_2021_86427_MOESM14_ESM.tiff]
